# Molecular Cloning, Transcriptional Profiling, Subcellular Localization, and miRNA-Binding Site Analysis of Six *SCL9* Genes in Poplar

**DOI:** 10.3390/plants10071338

**Published:** 2021-06-30

**Authors:** Meiqi Zhao, Lei Xuan, Haoran Qi, Tengfei Shen, Meng Xu

**Affiliations:** 1 Key Laboratory of Forest Genetics and Biotechnology of Ministry of Education, Co-Innovation Center for Sustainable Forestry in Southern China, Nanjing Forestry University, Nanjing 210037, China; Zmq@njfu.edu.cn (M.Z.); HaoranQi@njfu.edu.cn (H.Q.); stf.njfu@gmail.com (T.S.); 2 Institute of Botany, Jiangsu Province and Chinese Academy of Sciences (Nanjing Botanical Garden Mem, Sun Yat-Sen), Nanjing 210014, China; 13851991791@163.com

**Keywords:** ‘Nanlin895’ poplar, microRNA396, SCL9 subfamily, subcellular localization, adventitious root

## Abstract

The SCL9 subfamily is a key member of the GRAS family that regulates plant development and stress responses. Nevertheless, the functional role of these genes in the growth and development of poplar still unclear. Here, we reported the six *SCL9* genes, which were found to be differentially expressed during poplar adventitious root formation. The full-length sequences of *PeSCL9* genes of ‘Nanlin895’ poplar (*Populus deltoids* × *Populus euramericana*) were cloned by the RACE technique All *PeSCL9* genes lacked introns. RT-qPCR revealed that *PeSCL9* genes displayed a dynamic expression pattern in the adventitious root of poplar, according to RT-qPCR data. A series of comprehensive genes characteristics analysis were carried out for six genes by bioinformation. Meanwhile, transient expression analysis of the *Populus* protoplasts showed that all the PeSCL9 proteins were localized in the nucleus. In addition, the degradome and sRNA of ‘Nanlin895’ poplar in combination were used to predict miRNAs that regulate *PeSCL9*. It was found that miR396a and miR396c may affect *PeSCL9* expression via cleavage, which was further verified by a transient expression experiment in *Populus* protoplasts. Overall, the development of poplar adventitious root and other tissues was closely related to these six *SCL9* genes, and they serve as a starting point for further research into the mechanisms regulating poplar growth and development.

## 1. Introduction

Poplar is the common name for species of *Populus L.*, members of the Salicaceae, which are mainly distributed in the north temperate zone and cold temperate regions. Poplars are an important fast-growing industrial timber species in the mid-latitude plain, and these trees are also widely cultivated t worldwide due to their ecological and economic benefits [1]. On the other hand, poplar, is also a model tree for the study of basic forest biology and an ideal choice for the study of the molecular basis of complex forest traits. Poplar was the first woody perennial plant to undergo complete genome sequencing. It easily propagates asexually and grows rapidly. The genetic transformation system is relatively well established for poplar, and research on the functional genomics of these trees has a good foundation. The regulation of some key functional genes involved in growth and development was preliminarily revealed [2,3,4,5,6]. Some microRNAs (miRNAs) are involved in morphogenesis, secondary metabolism, and stress response via the targeted cleavage of protein-coding genes [7,8,9]. However, the progress to date is only the beginning, and there are still many basic issues to be further studied. Forest plants have more complex organogenesis and stress response mechanisms than herbaceous plants, and fine mapping and functional identification of the genes related to growth, development, and stress adaptability in these species still require further research.

The GRAS family is an ancient and unique transcription factor (TF) family in plants, and it is named for three genes: *gibberellic acid insensitive* (*GAI*) [10], *repressor of GA1-3 mutant* (*RGA*) [11] and *scarecrow* (*SCR*) [12]. GRAS proteins are generally composed of 400–770 amino acids that exhibit, a high degree of similarity at the carboxyl terminus. Leucine heptad repeat I (LHRI), VHIID, leucine heptad repeat II (LHRII), PFYRE and SAW are the five structural domains [13]. The nuclear localization signal (NLS) of most members of this family exists in the LHRI region, and the protein–DNA interactions are mediated by the VHIID motif [13]. The LXXLL motif, which exists in the LHRII region and mediates the binding of steroid receptor coactivator complexes to nuclear receptors [14]. Only the proline residues are highly conserved in the PFYRE, which is separated into three parts: P, FY, and RE. R-E, W-G, and W-W are three highly conserved amino acid residue pairs that compose the SAW motif [15,16,17]. The GRAS family participates in several aspects of plant organogenesis, morphogenesis, plant growth, development, and stress [18,19]. Based on the characteristics of the protein domains, the 33 Arabidopsis GRAS proteins could be divided into eight branches: LISCL (SCL9), HAM, PAT1, Scarecrow-like 3 (SCL3), DELLA, SCR, SHR, and LS [20]. *AtPAT1* is involved in the signal transduction of photosensitive pigments [21]. The DELLA protein is a central element in the GA signaling pathway that suppresses the signaling and development of plants by the negative regulation of GA signaling, while the SCL3 can weaken the DELLA repressor in the root endothelium, and then positively regulate the downstream pathway of GA signaling, which is participates in the development of roots and aboveground organs [22,23]. SCL3 competitively inhibits the interaction between DELLA and IDD (INDETERMINATE DOMAIN) protein, thereby inhibiting the expression of downstream genes [24]. SCR/SHR transcription factors regulate the radial development pattern of roots and the specialization of quiescent center properties [16,25,26,27]. LISCL (*Lilium longiflorum* Scarecrow-like) is a GRAS protein in lily. Its amino-terminal has strong transcriptional activation activity, and it is mainly expressed in the prophase of meiosis in anthers [28]. Notably, the number of genes belonging to the SCL9 subfamily is significantly greater than other subfamilies in most species [29], and the biological function of the SCL9 subfamily members are rarely reported. The overexpression of one gene encoding the SCL9 subfamily, *OsGRAS23*, can significantly improve the drought resistance and antioxidant capacity of rice [30]. Silencing of the *TaSCL14* gene resulted in slow plant growth, leaf senescence, and lower tolerance to photooxidative stress in wheat [31]. *AtSCL14* and glutathione GRX480/ROXY19 compete for combinations with the TGAII transcription factor to mediate the activation and inhibition of detoxification responses, respectively, in *Arabidopsis thaliana* [32,33,34]. Some small RNAs also interact with GRAS members to regulate growth and development [35,36,37,38].

The 106 GRAS TF members predicted in the poplar genome belong to 13 subfamilies [39], each of which is critical for poplar growth and development. To date, we have studied and reported the *HAM* [40], *PAT1* [41], *DELLA* [42], *SCR* and *SHR* [43] genes of the GRAS family, and verified some of their roles. The SCL9 subfamily is a great member of the GRAS family, which has been many studies on stress and development in diverse plants. However, there are relatively few reports on molecular biology and functions of the genes of the poplar SCL9 subfamily. In the present study, by screened from the genome sequencing information of poplar, we found that six *SCL9* genes had a different expression level during adventitious root development. We cloned and identified the full-length of six *PeSCL9* genes. Meanwhile, the *SCL9* genes and their protein sequences of ‘Nanlin895’ poplar (*P deltoids* × *euramericana*) were analyzed by bioinformatics approaches. The transcriptional regulation relationship between miR396a/c and *PeSCL9* was verified by combining the degradome with the *Populus* protoplast transient expression system. Our results indicated SCL9 subfamily had a function in the development in poplar, and supply the research blank. This research provides a basic understanding for further functional studies of *SCL9* genes in plants.

## 2. Results

### 2.1. Gene Structure and Promoter Analysis of SCL9 Genes

Six SCL9 family gene members that regulate the adventitious root development of *Populus* were screened out based on entire genome sequencing information of poplar and the expression profile of rooting poplar cuttings gene chip. The full-length sequences of six poplar *SCL9* branch genes were obtained by 3’ RACE and 5’ RACE procedures, and the genes were named *PeSCL9*, *PeSCL14-1*, *PeSCL14-2*, *PeSCL14-3*, *PeSCL30-1*, and *PeSCL30-2*, respectively, based on phylogenetic analysis. The basic characteristics of genes are enumerated in Table S1. The sequence length of genes ranged from 3005 bp (*PeSCL14-3*) to 2494 bp (*PeSCL30-1*). The length of the open reading frames (ORFs) of genes were within the scope of 1974 bp (*PeSCL30-1*) to 2280 bp (*PeSCL14-1*, *PeSCL14-2*, *PeSCL14-3*), and encoded polypeptides of 657–759 amino acids. The primary structures of the six proteins were predicted by ExPASy ProtParma. Their molecular weights were 74.82–85.52 kDa, and the theoretical isoelectric points were all less than seven, which indicates that the proteins were weakly acidic. The grand average of hydropathy (GRAVY) varied from −0.622 (*PeSCL30-2*) to −0.449 (*PeSCL9*). The secondary structures of the six proteins were tested by the self-optimized prediction method with alignment (SOPMA), and different results were obtained for each component. Among them, the contents of α helix structures and β corners in *PeSCL30-1* were higher and accounted for 42.77% and 4.11%, respectively. The content of extended chains in *PeSCL9* was 9.30%, and the content of random coils in *PeSCL14-3* was 49.67% (Additional File: Table S1). Moreover, genomic and cDNA sequence comparisons revealed that all six *SCL9* genes were all intron-free.

**Figure 1:**
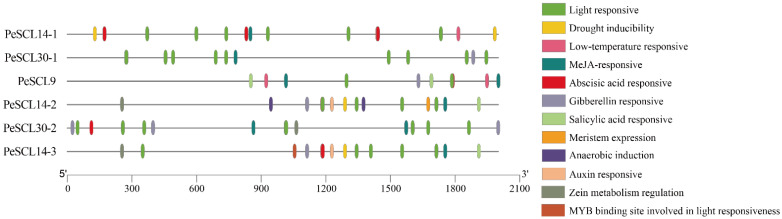
*Cis*-elements in the *PeSCL9* genes promoter regions. Different colors represent different *cis*-elements.

The *cis*-elements must be highlighted as crucial in the transcriptional regulation of gene expression. ‘Nanlin895’ poplar is closely related to *Populus deltoides*, and the genome was used as the reference genome to extract the 2000 bp upstream sequence of the start codon in the promoters of each gene from the *P. deltoides* genome (v.2.1). We used the PlantCare website to predict the *cis*-elements. A series of crucial *cis*-elements were found (Figure 1; Additional File: Table S2), including light response, auxin response, gibberellin response, abscisic acid response, MeJA response, and low-temperature response elements, which indicated that the *SCL9* genes regulate different biological processes.

### 2.2. Multiple Sequence Alignment, Phylogenetic Analysis, and Motif Composition of PeSCL9 Genes

**Figure 2:**
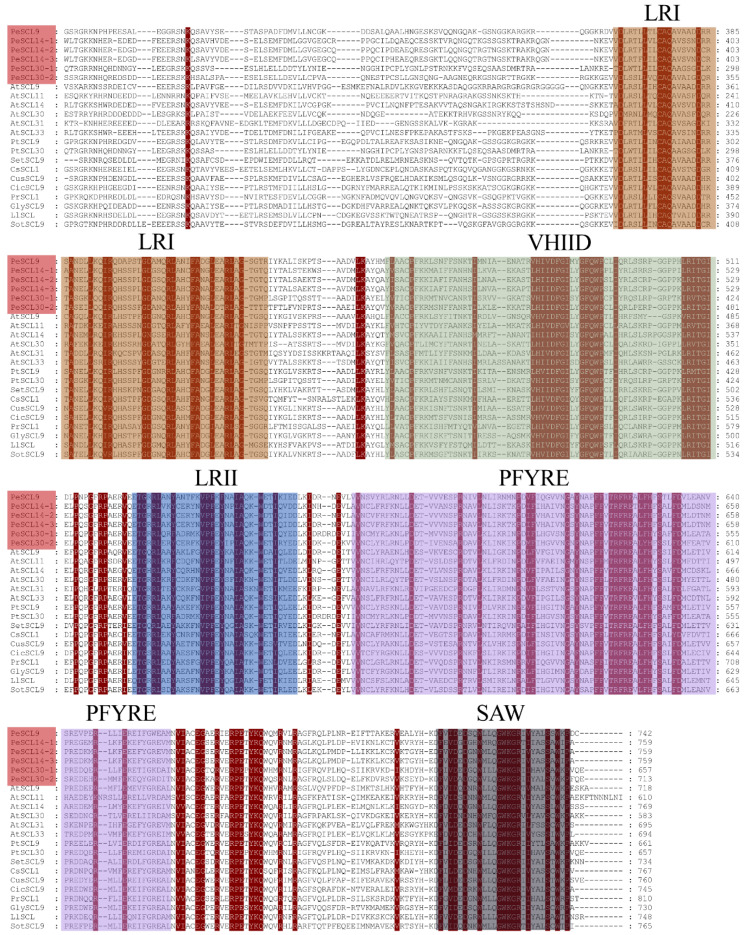
Sequence analysis of SCL9 proteins. ClustalX (v.2.1) alignment of the amino acid sequences of poplar SCL9, *Arabidopsis thaliana* SCL9, and other SCL9 proteins. The GRAS domains are indicated in different colors.

To further study PeSCL9 protein domains, we compared the GRAS domains of different species. The results revealed that the C-terminus of the proteins encoded by these six genes was consistent with the structure of 16 SCL9 subfamily proteins, and all contained LHRI, LHRII, VHIID, PFYRE and SAW domains specific to the GRAS family. No DELLA domain was found at the amino terminus (Figure 2), and these domains were distributed in the same order.

**Figure 3:**
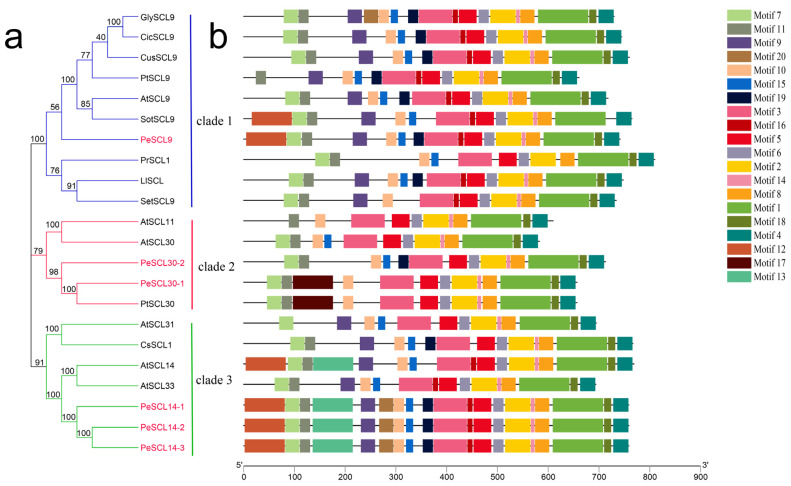
Phylogenetic relationship and gene structure of SCL9 proteins from different plant species. (**a**) The phylogenetic tree was generated based on the multiple sequence alignment of the 22 SCL9 proteins by using neighbor joining (NJ) method (bootstrap value = 1000). PeSCL9 proteins are marked in red; (**b**) conserved domains or motifs in the SCL9 proteins. The sequence information for each motif is provided in the Additional File: Table S3.

The phylogenetic relationships of PeSCL9 proteins were analyzed via alignment of the GRAS domain, represented by the GRAS domains of 16 SCL9 TFs from 10 species, including *Arabidopsis thaliana* and *Castanea sativa*. The results revealed that these six proteins were grouped into three clades. The SCL9 subfamily of PeSCL9 proteins and *Arabidopsis thaliana*, *Solanum tuberosum* and other species were clustered in Clade 1, and PeSCL30-1 and PeSCL30-2 are clustered in *Arabidopsis thaliana*. The SCL30 gene from *Populus trichocarpa* and other species were concentrated in Clade 2, and the homology of PeSCL14-1, PeSCL14-2, and PeSCL14-3 is very high. As in *Arabidopsis thaliana*, AtSCL14, AtSCL33 and other proteins were clustered in Clade 3 (Figure 3a), and the results were essentially identical to the phylogenetic analysis, with differences between groups but similarities within groups. The motifs of 22 SCL9 proteins were examined by MEME online to learn more about the SCL9 proteins characteristic region. Twenty distinct MEME motifs (designated motifs 1–20) were discovered (Figure 3b; Additional File: Table S3). The SCL9 proteins in the same evolutionary lineage generally had the same motif composition (e.g., PeSCL30-1 and PtSCL30), which suggested that similar functions are the result of genes from the same evolutionary lineage.

### 2.3. Subcellular Localization of SCL9 Proteins

**Figure 4:**
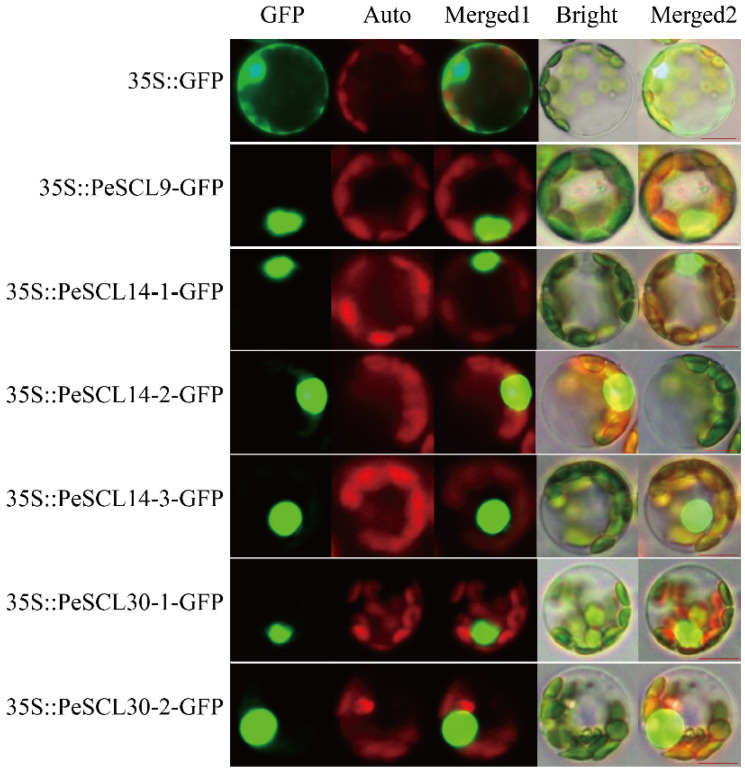
Subcellular localization of SCL9 branch genes in *Populus* mesophyll protoplasts. Green fluorescence protein (GFP), chlorophyll autofluorescence (Auto), Merged 1, bright and Merged 2 images are shown. Scale bar = 10 μm. The 35S::GFP fusion was used as a positive control protein and was detected in the nucleus and cytoplasm in *Populus* protoplasts.

To understand the function of SCL9 proteins, we further analyzed the subcellular localization of six *PeSCL9* genes using GFP as a tag. The positive control spread throughout entire cells, and the six fusion proteins in the SCL9 branch all produced green fluorescence in the nuclear region, which indicated that the proteins encoded by the six genes in this study were all localized in the nucleus. These findings suggest that these genes have a transcriptional regulatory function in the growth of poplar (Figure 4).

### 2.4. Expression Patterns of PeSCL9 Genes

**Figure 5:**
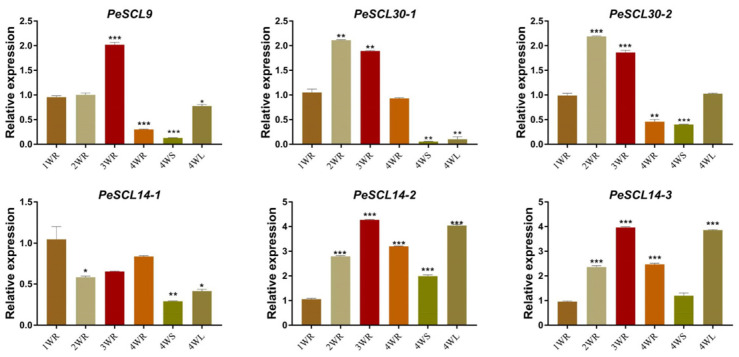
Expression patterns of SCL9 subfamily genes at different time points and tissues of *Populus*. Temporal and spatial expression patterns of poplar *SCL9* genes by real-time RT-PCR. The *Y*-axis represents relative quantitation and the *X*-axis represents different time points/tissues. The error bars represent the mean ± SE, where *n* = 3. Statistical significance of differences was determined by the Student’s *t* test (*p < 0.05, ** *p* < 0.01, *** *p* < 0.001, compare to 1 WR).

To verify the expression of *PeSCL9* genes during the development of *Populus*, RT-qPCR was performed to analyze the expression of genes in diverse tissues and organs of poplar and at different stages of root development (Figure 5). Intriguingly, the expression of *PeSCL30-1* was almost nonexistent in leaves and stems after four weeks, and the expression of *PeSCL14-1* was highest in roots. The expression levels of the other four genes were the highest in leaves and the lowest in stems. However, the overall expression level of *PeSCL14-1* was lower than the other genes. In addition, these genes also showed diverse expression trends at different developmental stages of roots. The expression trend of *PeSCL14-1* gene in roots at four weekly developmental stages (1 W, 2 W, 3 W, 4 W) was “decreased first and then increased”, with the highest expression level at 1 W. The remaining five genes were “increased first and then decreased”. The expression of *PeSCL30-1* and *PeSCL30-2* was the highest at 2 W, and the expression of *PeSCL9*, *PeSCL14-2* and *PeSCL14-3* was the highest at 3 W. These results indicated that these six genes showed different expression patterns in different tissues and different developmental stages of poplar roots.

### 2.5. MiR396a/c-Directed Targeting of PeSCL9 Genes in Populus

**Figure 6:**
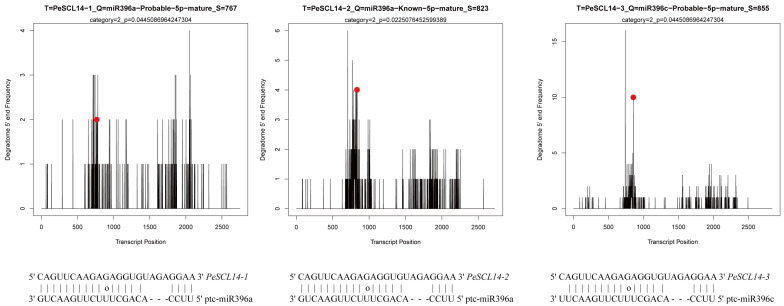
Target plots (t-plots) of representative Pe-miRNA targets confirmed by degradome sequencing.

According to the sRNA database of ‘Nanlin895’ poplar, we used the psRNATarget website to search for potential miRNAs that may regulate *PeSCL9* genes. We identified 36 potential miRNAs that may regulate target genes by cleavage using a mismatch score of 4.0 (Additional File: Table S4). The cleavage sites of PeSCL9 mRNAs were further identified based on the degradome from our laboratory. Cleaveland4 was used to predict the cleavage sites. To improve the prediction accuracy, only miRNAs with category scores of 0–2.0 were considered candidate miRNAs. The results showed that miR396a could target *PeSCL14-1* and *PeSCL14-2*, and miR396c regulate *PeSCL14-3* (Figure 6). The cleavage sites were at 767, 823 and 855, respectively. Remarkably, mature miR396a-5p and miR396c-5p were highly homologous, which suggests regulation of the same target genes.

**Figure 7:**
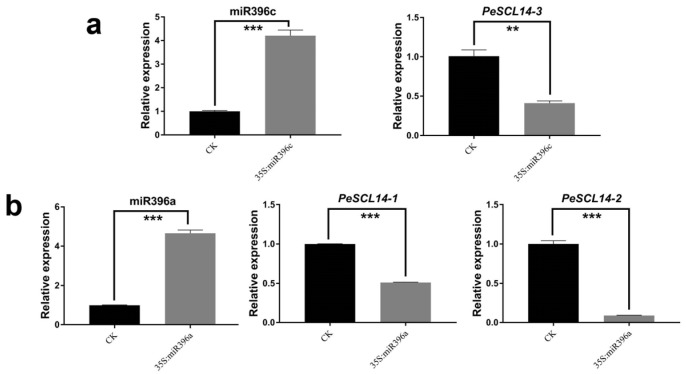
The expression pattern of miR396 and its target gene *PeSCL9* in *Populus* protoplasts. The *Y*-axis represents relative quantitation and the *X*-axis represents different expression vectors. (**a**) Overexpress of miR396c leads to a decrease in the expression of *PeSCL14-3*; and (**b**) overexpress of miR396a leads to a decrease in the expression of *PeSCL14-1* and *PeSCL14-2*. The error bars represent the mean ± SE, where *n* = 3. Statistical significance of differences was determined by the Student’s *t* test (** *p* < 0.01, *** *p* < 0.001, compared to control).

To detect whether the miR396a/c target genes were consistent with the degradome sequencing results (Figure 6), we studied miR396 regulation of target genes by the transient expression system of poplar [44] and measured the expression levels of three target genes after the transient overexpression of miR396a/c by RT-qPCR. The control check (CK) was the normal protoplast without any plasmid transfer. The expression levels of miR396a in protoplasts transfected with 35S::miR396a and 35S::miR396c were significantly higher than the CK. As expected, target genes expression were reduced in 35S::miR396a and 35S::miR396c compared to the control. Strikingly, transient overexpression of miR396a reduced the expression of *PeSCL14-2* by approximately 10 fold (Figure 7). These results confirmed that miR396 targeted *PeSCL9* genes.

## 3. Discussion

GRAS is a specific and widespread TF family in plants, and is involved in many processes of plant growth and development. The genome-wide identification of GRAS family members of diverse species has been gradually completed. The amount of GRAS genes in *Populus* is approximately two to three times the number in rice and *Arabidopsis thaliana*. Notably, 22 percent of GRAS genes in *Populus* are produced by the combined mechanism of tandem and segmental replications, compared to just eight percent in *Arabidopsis thaliana* and rice, which suggests that this distinction may underlie the fast proliferation of the GRAS family in *Populus* [39]. Species genetic differences and the rate of evolution are linked to a variety of causes, and environmental changes can also have a strong impact on plants [45]. Only a small number of GRAS genes in poplar have been studied [46,47]. We previously reported that HAM [40], SHR/SCR [43], DELLA [42], and PAT1 [41] transcription factors may be involved in the growth and development of adventitious root, stems, and leaves of poplar. SCL9 is a member of the GRAS family, and it was the first GRAS protein to be implicated in the direct control of specific gene expression. The amino terminus of the *LISCL* gene, a new GRAS gene in lily, bears no resemblance to the SCR protein, and neither molecule has the DELLA domain [28]. Therefore, these authors speculated that the *LISCL* gene had specific functions, and demonstrated that the N-terminus of the LISCL protein had significant transcriptional activation ability and played a role in anther meiosis. Six *SCL9* genes were cloned from the ‘Nanlin895’ poplar to investigate the role of the SCL9 subfamily in poplar. Sequence alignment showed that these genes were similar to the SCL9 subfamily of *Arabidopsis thaliana* and *Castanea sativa*, and contained conserved GRAS domains [13]. Phylogenetic analysis divided these six genes into three clades. In the meanwhile, there appeared to be a similar motif structure in each clade, which indicated the same function in plant development. For example, previous studies have shown that *CsSCL1* and *PrSCL1* genes are mainly expressed in the roots, and they perform key functions in the primary stage of adventitious root formation. GRAS proteins from these two distant tree species exhibit similar sequences and functions, which indicated that these two proteins have conserved functions in the process of adventitious root formation [48]. *AtSCL14* ameliorates the stress of photooxidation [49]. The *PeSCL14-1*, *PeSCL14-2*, and *PeSCL14-3* genes were highly homologous with *AtSCL14* and *CsSCL1*, which suggests their involvement in the establishment of the poplar root meristem and the detoxification reaction. The six genes cloned in the present study had the same domain as LISCL, which suggests their involvement in the meiosis process in poplar.

*Cis*-elements are essential regulators of plant transcription in response to abiotic stress and plant hormone responses [50,51]. Previous studies found that the domain of LISCL was related to transcriptional activation or co-activation in response to various signals [52]. The present study examined *cis*-elements in the promoter region of the *PeSCL9* genes. At least one *cis*-element related to plant hormones was found in each gene (Figure 3). The *PeSCL14-2* and *PeSCL14-3* genes had five hormone-responsive elements, which were highly homologous and suggest that these genes play key roles in the response of poplar to hormones. Only *PeSCL14-1* and *PeSCL9* were involved in the low-temperature response. GRAS proteins, such as SIGRAS40 [53], OsGRAS23 [30], and BnGRAS25 [54], play roles in plant and abiotic stresses. Therefore, these genes likely regulate abiotic stress responses and hormones. In addition, our subcellular localization results showed that the proteins encoded by six *SCL9* genes were all localized in the nucleus, which suggests a role for these genes in transcriptional regulation.

Previous studies demonstrated that *SCL9* genes were involved in adventitious root formation and other regulatory processes [55]. Among them, *ZmGRAS25* is highly expressed in maize primary roots and may be involved in the formation of the maize root system [56]. The expression levels of the *SCL9* genes were the highest in the roots of *Tartary buckwheat* [17]. The results suggest that the SCL9 subfamily may play a very important role in the growth and development of plant roots. We hypothesized that these six *SCL9* genes were involved in adventitious root formation. Therefore, RT-qPCR was used to measure the expression of these six genes in diverse tissues of poplar. The expression trends of four genes were consistent in growing roots, stems and leaves for four weeks, but *PeSCL30-1* and *PeSCL14-1* expression were not consistent. The expression levels of these genes were higher in leaves and the lowest in stems. Measurement of the expression levels of these genes at different periods of root development showed that the expression levels of *PeSCL14-1* were high in the early stage (1 WR) and maturation stage (4 WR) of adventitious root development. The remaining genes were expressed during early and mid-term root development, with expression peaking at two to three weeks then decreasing.

MiRNAs play core roles in embryogenesis, root and leaf development, and the responses to biotic and abiotic stress [57,58]. MiR396 is a highly conserved family of plant miRNAs that are found in all land plants [59]. MiR396 plays important role in many aspects of plants. MiR396a-3p/5p and miR396b are mainly expressed in fruits and make fruits larger and play an important role in fruit yield and crop improvement [60]. MiR159 and miR396 regulate floral characteristics and timing in dicotyledons and monocotyledons [61]. Meanwhile, miR396 inhibits the expression of growth-regulating factors (GRFs) to regulate the transition of root stem cells to transitional proliferating cells, and it participates in the process of root development [62]. The present study examined the relationship between miR396a/c and *PeSCL9* genes and found two miRNAs that may regulate the expression of three *PeSCL9* genes by cleavage based on analysis of the sRNA and degradome data. MIR396a matched *PeSCL14-1* and *PeSCL14-2*, and miR396c matched *PeSCL14-3*. To further confirm the regulation of *PeSCL9* and miR396a/c, the expression levels of target genes were analyzed after transient overexpression of miR396a/c, and the results indicated a decrease in all of the target genes to varying degrees. Therefore, we speculated that miR396 participated in plant growth and development via the cleavage of *PeSCL9* genes.

*Populus* is the most widely distributed tree. It is an economically important tree. Some studies used transcriptome sequencing, genome resequencing and bioinformatics methods to identify genes, which are associated with a lignin pathway. The associated genetic strategy was used to analyze 124 additional SNP sites on the formation of wood formation, dominant and upper genetic effects, and constructed lncRNA-miRNA-mRNA genetic regulation network [63]. In addition, a total of 462 SNPs were found from poplar, which will be very useful for assessment of breeding populations [64]. In this study, we found miR396 decreased the expression level of *SCL9* genes, which indicated miRNAs play a pivotal role in the regulation at the transcriptional level through genetic interaction.

In present, the specific function of SCL9 subfamily in plants is still in basic research. With the development of plant genomics, we should make full use of the information to solve the existing biological problems, so as to achieve the purpose of improving the traits of tree species [65], gene editing and transgenics can also be used to study the function of genes. In addition, miRNA, as an important non-coding RNA in a class of organisms, is a key part of the gene regulation network. We can study the interaction with target genes by means of mutated miRNA binding sites, dual-luciferase reporter assay system, 5’-RLM-RACE, etc.

## 4. Materials and Methods

### 4.1. Plant Materials and Growth Conditions

All sampled plantlets of ‘Nanlin895’ poplar were placed in a light incubator (16/8 h light/dark, temperature of 25/18 °C, 70% relative humidity) on Murashige and Skoog (MS) medium (pH 5.8). The roots at one week (1 WR), two weeks (2 WR), three weeks (3 WR) and four weeks (4 WR), the stems (4 WS), and the leaves (4 WL) were used as tissue materials. The samples were stored at −80 °C prior to RNA extraction to determine the degree of expression in various organs and developmental periods. The leaves of tissue culture seedlings grown for 40 days were used for protoplast separation and transient transformation.

### 4.2. RNA Isolation and Gene Expression Analysis

Total RNA was isolated from poplar using RNeasy Plant Mini Kit (Qiagen, Hilden, Germany) according to the manufacturer’s instructions, and the RNA was purified by DNase I (Qiagen, Hilden, Germany). The concentration, purity (A260/280 = 1.8–2.1) and integrity of total RNA were measured using a Nanodrop 2000c spectrophotometer (Thermo Fisher, Waltham, MA, USA) and 1% agarose gel electrophoresis, respectively. PrimeScript^TM^ RT Master Mix was used to reverse-transcribe cDNA (Takara, Otsu, Japan). According to the manufacturer’s instructions of the Mir-X miRNA First-strand Synthesis Kit (Takara, Otsu, Japan), 1 µg of miRNA and other RNA molecules were transformed into cDNA. Real-time quantitative primers were designed using Oligo7 software. The *elongation factor 1 alpha* (*EF1α*) gene (GenBank Accession Number: AJ536671) [66] was used for normalization. Real-time PCR was conducted using a ViiA^TM^ 7 Real-time PCR System (Applied Biosystems, New York, NY, USA) with PowerUP^TM^ SYBR^TM^ Green Master Mix (Thermo Fisher, Waltham, MA, USA). The real-time PCR system protocol was followed. The following reaction procedure was used: UDG activation at 50 °C for 2 min, Dual-Lock™ DNA polymerase at 95 °C for 2 min, 40 cycles of denaturation at 95 °C for 15 s, and annealing/extension at 60 °C for 1 min. The dissociation curve conditions (melt curve stage) were set at 95 °C for 15 s, 60 °C for 1 min, and 95 °C for 15 s. The specific mRQ3’ primers and miRNA mature sequences (miR396a: UUCCACAGCUUUCUUGAACUG, miR396c: UUCCACAGCUUUCUUGAACUU) were used for real-time PCR. The reference genes and reaction system are described above. Each experiment had three technical replicates, and the relative quantitative analysis was performed using the 2^−^^△△Ct^ method. All of the primer sequences are listed in Supplementary Table S5 (Additional File: Table S5).

### 4.3. Gene Cloning of Full-Length PeSCL9 Genes

Primers for 3’RACE and 5’RACE PCR amplification were constructed using the probe sequence from the poplar genome chip. The full-length sequence was amplified by nested PCR according to the operation instructions for the 3’ Full Race Kit and 5’ Full Race Kit (Takara, Otsu, Japan). The target fragment was recovered and purified using an Axygen gel extraction kit (Axygen, Central Avenue, CA, USA) after the PCR product was detected by 1% agarose gel electrophoresis. The isolated fragment was connected to the pMD19-T vector (Takara, Otsu, Japan) and transformed into TOP10 (Takara, Otsu, Japan). The positive clones were screened and sequenced for analysis. The 3’ RACE and 5’ RACE sequences were matched and spliced to obtain the full-length gene sequence. The same primers were used to amplify the predicted ORFs and genomic DNA sequences through the high-fidelity KOD enzyme (Takara, Otsu, Japan), and then the number and location of introns were determined by comparison. Supplementary Table S5 lists the primer sequences utilized in this investigation (Additional File: Table S5).

### 4.4. Genes Sequence Analysis

The NCBI ORF Finder (https://www.ncbi.nlm.nih.gov/orffinder/ accessed on 5 October 2020) was used to predict the ORF sequences. The basic characteristics of the genes and amino acid composition of the proteins were analyzed by the ExPASy ProtParma website (https://web.expasy.org/protparam accessed on 5 October 2020). The online SOPMA program (https://npsa-prabi.ibcp.fr/cgi-bin/npsa_autmat.pl?page=/NPSA/npsa_sopma.html accessed on 5 October 2020) was used to predict the secondary structure of amino acid sequences in October 2020. The GRAS domain was inferred from the Pfam database in October 2020 (http://pfam.xfam.org/ accessed on 12 March 2021) and the E-value cut-off was 1e-5. The latest protein file of the *Populus deltoides* genome was downloaded from Phytozome (v.12.1) (https://phytozome.jgi.doe.gov/pz/portal.html accessed on 12 March 2021) and the 2000 bp sequences upstream of six genes were extracted in TBtools software (v.1.0971) [67] and submitted to the PlantCARE online database (http://bioinformatics.psb.ugent.be/webtools/plantcare/HTML accessed on 16 March 2021) to predict *cis*-elements in the promoter region in March 2021 [68]. The TBtools program was used to draw the *cis*-elements of genes. The Plant Transcription Factor Database (http://planttfdb.gao-lab.org/ accessed on 19 March 2021) was used to obtain the domain sequences of the GRAS proteins from *Arabidopsis thaliana*, *Castanea sativ*, and other plants. The protein sequences of the six genes discovered were compared to other plants using ClustalX (v.2.1) software. Then, MEGA X software was used to conduct phylogenetic analyses in November 2020, with the neighbor-joining (NJ) algorithm and 1000 bootstrap replicates [69]. GeneDoc (www.psc.edu/biomed/genedoc accessed on 18 November 2020) software was used to manually adjust the amino acid sequence in the SCL9 domain. PeSCL9 proteins were subjected to conserved base sequence analysis by MEME in November 2020 (http://meme.nbcr.net/meme/cgibin/meme.cgi accessed on 18 November 2020) with default parameters, and the largest base sequence parameters of 20 [70]; the results were visualized using TBtools in March 2021. The psRNATarget website (http://plantgrn.noble.org/psRNATarget/ accessed on 13 December 2020) was used to predict miRNAs that may regulate *SCL9* genes in December 2020. To find potentially cleaved targets, the CleaveLand4 (v4.5) pipeline was used [71]. All alignments with a category ≤ 2 and a *p*-values ≤ 0.05 were considered candidate targets. The degradome and sRNA data were obtained from the laboratory (Accession Numbers PRJNA498391 and PRJNA498400) [44].

### 4.5. Expression Vector Construction

The expression vectors were constructed according to the Gateway Technology Protocol (Invitrogen, Carlsbad, CA, USA). For transient expression assays, the coding region of each gene, without the stop codons, was cloned into pCR^TM^8/GW/TOPO (Invitrogen, Carlsbad, CA, USA) to generate an entry clone. Then we transformed into competent *E.coli* cells, and choose a positive transformant and isolate plasmid DNA. We generated an expression vector by performing an LR recombination reaction between the entry clone and P2GWF7.0 vector, with a C-terminus of the green fluorescent protein (GFP) under the control of CaMV 35S promoter. The mature sequences of miR396a and miR396c were obtained from the sRNA data. Specific primers (Table S5) were used to amplify the precursor sequence of miR396a/c from the genomic DNA. The sequences were cloned into the overexpression vector pH35GS under the control of the 35S promoter using the ClonExpress II One Step Cloning Kit (Vazyme, Nan Jing, China). The vectors were then transformed into competent *E.coli* cells and used for the next experiments.

### 4.6. Transient Expression Assays

Protoplast isolation and polyethylene glycol (PEG)-mediated transformation were performed using the method of our laboratory, with minor changes [72]. Briefly, the 100 µg 35S::miR396a/c plasmid was transferred into the 1 mL protoplast, and cultured at 20–25 °C in darkness for 16 h. The experiment was repeated three times. Total RNA was extracted by the method described above for analysis of gene expression levels. At the same time, the constructed transient expression vector was transformed into protoplasts. After 16 h of dark culture, the GFP fluorescence signal was observed to produce 509 nm green fluorescence under excitation with 488 nm blue light using a fluorescence microscope (Carl Zeiss, Jena, Germany). The data represent three independent experiments.

### 4.7. Statistical Analysis

All experiments were repeated at least three times. The RT-qPCR raw data were calculated according to the 2^−ΔΔCt^ method. Statistical significance of differences was compared by the Student’s *t* test. “*” for *p* < 0.05; “**” for *p* < 0.01; “***” for *p* < 0.001 were known as significant. Data were plotted as means ± SE. The error bars represent the mean ± SE. GraphPad prism software (v.7.0) was used to generate graphs.

## 5. Conclusions

The present study analyzed, the role of the SCL9 subfamily in adventitious root of *Populus*. The results enrich our knowledge of the GRAS family of transcription factors in poplar, and further exploring SCL9, an important member of the GRAS transcription factor family, to provide a reference for research on the function of plant growth and development. Future studies on the poplar adventitious root formation mechanism will provide further theoretical support.

## Data Availability

The latest protein sequences file of *Populus deltoides* genome was downloaded from Phytozome (v.12.1) in March 2021 (https://phytozome.jgi.doe.gov/pz/portal.html). The degradome were deposited in the Sequence Read Archive (SRA) database of the NCBI under accession number PRJNA498391. The sRNA-seq data submitted to SRA database under accession number PRJNA498400.

## Supplementary Materials

The following are available online at https://www.mdpi.com/article/10.3390/plants10071338/s1, Table S1: analysis of the protein structure encoded by six SCL9 branch genes in Populus, Table S2: Cis-elements analyses of the PeSCL9 genes promoter regions, Table S3: analyses the motifs in PeSCL9 proteins from the MEME website, Table S4: comparison of PeSCL9 with miRNA matching sites of ‘Nanlin895’ poplar, Table S5: primer sequences.
